# Facilitators and barriers to the implementation of DIP payment methodology reform in a public hospital in Guangzhou: a qualitative study based on the implementation of the meta-framework for research (CFIR) framework

**DOI:** 10.3389/fpubh.2025.1569855

**Published:** 2025-05-30

**Authors:** Jinghui Chang, Siyi Chen, Anqi Li, Xixi Yang, Hualian Luo, Maidina Yilamu, Bingqian Fu, Nuoyan Xu, Jing Liu, Huishu Tian

**Affiliations:** ^1^School of Health Management, Southern Medical University, Guangzhou, China; ^2^Guangzhou First People’s Hospital, Guangzhou, China

**Keywords:** public hospitals, internal management, DIP Medicare payment methodology, meta-framework for implementation research (CFIR) framework, public health policy

## Abstract

**Background:**

The Diagnosis-Intervention Packet (DIP), a medical insurance payment management system utilizing big data, has been piloted in 12 cities by the National Healthcare Security Administration in China starting in 2021. Guangzhou is one of the pilot cities, and it has demonstrated significant success in the DIP payment reform, with its practical experience being affirmed and promoted by the National Health Department. In this study, researchers conducted field visits to a public hospital in Guangzhou to understand the internal responses to the DIP reform and the cognitive attitudes of relevant personnel. The analysis of the positive and negative factors affecting the implementation of the reform and the proposed measures to optimize internal hospital management are expected to provide practical evidence for the implementation of DIP payment reform in other public hospitals.

**Methods:**

This study develops an interview guide based on the Consolidated Framework for Implementation Research (CFIR) and conducts one-on-one semi-structured interviews offline with personnel from a Grade A tertiary public hospital in Guangzhou. Employing rapid qualitative analysis techniques and utilizing NVivo 14.0 for coding CFIR-structured texts related to implementation, the study integrates five dimensions: innovation, inner and outer context, individuals, and the implementation process. It identifies factors that facilitate and hinder the implementation of the Diagnosis-Intervention Packet (DIP) payment reform, thereby proposing optimized internal management strategies for public hospitals to cope with DIP payment reforms.

**Discussion:**

This study will provide significant insights for optimizing the internal management of public hospitals in the context of DIP payment reform. It offers a reference for optimizing internal management in tertiary public hospitals in China, aiming to achieve standardized, healthy, collaborative, and high-quality development.

## Background

The stable and efficient operation of medical insurance funds is crucial for as well as. Changes significantly on allocating and coordinating medical resources and services, and changes in medical insurance payment methods significantly impact the efficiency of medical insurance fund utilization (1) Therefore, the reform of medical insurance payment methods occupies an important position in the medical reform process (2). Since 2009, China has been exploring the implementation of Diagnosis Related Group (DRG) payment reform, progressively eliminating the excessive medical practices associated with the traditional fee-for-service model (3). In 2019, the National Health Department, in collaboration, issued the technical specifications and grouping schemes for DRG pilot programs, designating 30 cities as national pilot cities for DRG payment (4).

China’s exploration of localized medical cost containment strategies began in 2018, based on authentic domestic data. It objectively summarized the characteristics of various diseases and their treatment paradigms and, from these common features, established a Diagnosis-Intervention Packet (DIP) system that aligns with the current state of medical services in China. Gradually, a medical payment framework was constructed that supports a “total budget for normal growth; disease category points to guide rational treatment, payment reflects incentive mechanisms, and intelligent monitoring to strengthen process management” (5). Consequently, in 2020, the National Medical Insurance Administration issued the “Regional Point Method Total Budget and DIP Pilot Work Plan,” officially launching DIP pilot programs in 71 cities nationwide. To date, the National Medical Insurance Administration has published seven policy documents, emphasizing from the national health strategy level the construction of a “practical and efficient medical insurance payment mechanism” adapted to the national conditions.

In 2021, the National Healthcare Security Administration, guided by developmental objectives, comprehensively and concurrently advanced DRG/DIP payment methodologies reform, issuing the “Three-Year Action Plan for DRG/DIP Payment Method Reform” (6, 7). It proposed that by 2022–2024, the reform tasks for DRG/DIP payment methods would be fully completed, thereby promoting high-quality development in medical insurance. By the end of 2024, all regions under the unified management had initiated the reform of DRG/DIP payment methodologies (8). By the end of 2025, the DRG/DIP payment method is expected to cover all eligible medical institutions providing inpatient services nationwide, achieving comprehensive coverage across four aspects: unified regions, medical institutions, disease groupings, and medical insurance funds (9).

By the strategic planning of the National Healthcare Security Administration, regions are advancing medical insurance payment reforms in a phased and batched manner, implementing them steadily through a combination of national unified deployment and local practice. Observations from the practices in various provinces and cities indicate that the DRG/DIP payment system has achieved certain effects. By the end of 2021, it had entered the actual payment phase, which can essentially ensure that medical institutions retain a certain surplus (10). Taking Zhejiang Province, which entered the actual payment phase earlier, as an example, the province has included inpatient costs across the entire province, encompassing all demographics and aspects of diagnosis and treatment within the scope of DRG payment reform (11). The DIP reform has also yielded preliminary results: in pilot cities of Guangdong, Jiangsu, Fujian, and Shandong provinces, there has been a significant reduction in the growth rate of medical expenses and average inpatient costs, with the growth rate of medical insurance payments for inpatient costs decreasing by up to 4.8%, thereby alleviating the pressure on fund expenditures to a certain extent (12).

Guangzhou, with the richest medical resources in southern China, with the number of top-tier hospitals ranking only after Beijing and Shanghai nationwide. As one of the first pilot cities for the Diagnosis-Intervention Packet (DIP) payment reform, since January 2018, it has fully implemented a big data-based case-based payment system, covered 334 medical institutions and saved 1.1 billion yuan in medical insurance costs in that year alone, establishing an integrated payment supervision mechanism of “retaining surpluses and sharing deficits” (13). In 2020, as a national advanced pilot for DIP, the National Healthcare Security Administration promoted the “Guangzhou Experience” to the national level. By 2023, the average inpatient cost and out-of-pocket expenses in Guangzhou had decreased by over 10%, reducing unnecessary hospitalizations by 56,000 person-times annually, and the average number of hospital stays per patient declined by 5.8%. This effectively controlled the irrational increase in medical expenses and promoted the rational allocation of medical resources (14). The DIP payment reform in Guangzhou, Guangdong Province, has covered all designated inpatient hospitals, benefiting 14 million insured individuals in the city.

The effectiveness of the Diagnosis-Intervention Packet (DIP) payment reform has garnered widespread attention, as it not only meets the basic medical needs of the population but also alleviates the financial burden on medical institutions (15). Many scholars believe that DIP payment, as an indigenous innovation in the management model of medical insurance funds, will effectively address issues such as excessive medical treatment and the extensive development of hospitals (16). A bundled pricing approach achieves precise pricing and reasonable compensation for medical services, playing a positive role in controlling the irrational increase in medical expenses (17). However, the implementation of DIP still faces certain obstacles. The shift in payment methods and the management pressure due to overly detailed categorization have increased the operational burden on hospitals. Under the pressure of reform targets (18), profit-driven behaviors such as patient referral and conservative treatment are hard to avoid. Weak support from electronic medical record platforms, benefit disparities between disease categories, and insufficient awareness among medical staff and patients constrain the promotion and implementation of DIP (19). Profit-driven behaviors such as patient referral and conservative treatment are hard to avoid. Weak support from electronic medical record platforms, benefit disparities between disease categories, and insufficient awareness among medical staff and patients constrain the promotion and implementation of DIP.

Against the backdrop of medical reform, scholars often begin their investigation with the operational effects of the Diagnosis-Intervention Packet (DIP) reform on medical institutions, examining its impact on aspects such as medical quality, medical expenses, and hospital stay duration. From a research perspective, domestic and international scholars focus on policy orientation, system construction, and multi-stakeholder collaboration (20), emphasizing macro-level policy analysis. In contrast, studies on individual policy cognition and evaluation at the implementers’ level are relatively scarce (21). However, the in-depth implementation of DIP requires a combined qualitative approach from both macro and individual perspectives to better explore how health policies can be effectively implemented within hospitals.

The Consolidated Framework for Implementation Research (CFIR), initially introduced in 2009, comprises five dimensions and 39 domains that influence the implementation process and outcomes (22). After a decade and a half of application and evolution, in 2022, the CFIR development team updated the framework in response to feedback from research scholars, resulting in CFIR 2.0. This updated version expanded the framework by adding 21 new constructs and 19 secondary constructs. It refined some of the existing constructs and their definitions (23, 24), enhancing their utility and applicability. Worldwide, in the assessment of healthcare service quality, the CFIR framework is extensively applied in practical investigations across various fields such as nursing implementation, screening interventions, clinical decision-making, medical platform evaluation, and health analytics, promoting a more profound integration of implementation science theory with empirical research (25, 26).

The CFIR used in this study provides a theoretical and methodological basis for analyzing DIP payment reform. Compared to frameworks such as RE-AIM or PARIHS, CFIR provides a more structured approach to assessing the contextual, behavioral, and organizational dynamics that influence implementation outcomes (22, 27). When involving multiple levels of participants in the policy, institutional, and clinical arenas, CFIR is better suited than the Theoretical Domains Framework (TDF) to study complex policy reforms, capturing the interplay between policy characteristics, institutional context, and individual behaviors that are critical to understanding the real-world challenges of healthcare system reforms (28) CFIR’s five-domain structural design allows for a better layered understanding of how reforms are perceived (innovations), experienced at the institutional and inter-institutional levels (internal and external environments), implemented by individuals, and how the adaptation process takes shape - dimensions that are highly relevant to the context of top-down policy diffusion and local adaptation in China. In addition, CFIR has been successfully applied to implementation studies of DRG-based payment reforms in multiple countries, enhancing its cross-country and cross-system applicability. For example, in South Korea, Kim et al. (29) used CFIR to assess how hospitals are responding to bundled DRG-based payments, identifying organizational leadership and system infrastructure as key enablers. In Germany, Linn et al. (30) utilized CFIR to explore the implementation of digital health in a DRG environment. Similarly, in France, Dufour et al. (31) used CFIR to examine behavioral responses to performance-based hospital grants.

By adopting CFIR in the context of China’s DIP reforms, this study can serve to fill a comparative gap in how developing health systems respond to complex payment reforms. CFIR’s cross-country relevance and its flexibility in integrating micro- and macro-implementation determinants strengthen its applicability in this study.

Although there has been a great deal of research on the institutional design and macro-effectiveness (e.g., cost control, efficiency improvement) of the DIP payment reform, most of the literature focuses on the macro-policy level or the analysis of quantitative economic indicators, and there is a lack of qualitative research on the complexity of the healthcare reform in the process of hospitalization, the resistance and adaptive mechanism of the implementation of the healthcare reform, especially from the perspectives of the main actors of the implementation -- hospital administrators and clinical staff. However, most of the literature focuses on the macro policy level or quantitative economic indicators to analyze the complexity, resistance and adaptation mechanism of the implementation of the reform in the frontline of hospitals, especially from the perspective of the implementation subjects - hospital administrators and clinical staff. This “implementation gap” limits our in-depth understanding of the actual operation mechanism of DIP reform and is not conducive to the identification and optimization of system adaptation problems at the micro level.

Therefore, using CFIR as a theoretical framework, researchers conducted semi-structured interviews with administrators, clinical department heads, and clinical technicians. Unlike studies that rely on large samples of data or disease-specific analyses, this type of holistic frame analysis and coding comparison for a single sample of healthcare organizations can reveal facilitators and major barriers to policy implementation in hospitals. In contrast to questionnaires and quantitative data analysis methods, interviews can provide insights into the perceptions and feedback of hospital insiders on the current status of DIP payment reform, and further explore the major issues faced by this tertiary care public hospital in the implementation of DIP payment reform.

This study focuses on the internal implementation process of DIP reform in a public tertiary hospital, aiming to answer the following core research questions:

- How do medical staff and administrators perceive and adapt to the DIP payment reform?
- What are the organizational, process, and individual challenges and facilitators faced by hospitals in the implementation of DIP reform?
- How can recommendations be made to optimize the path of reform implementation from the perspective of the implementers?

Through semi-structured interviews and framework-oriented coding analysis, this research attempts to fill the gap of the lack of micro-mechanisms explored in existing studies at the implementer level and provides empirical insights and strategic references for the policy implementation of DIP reform and the continuous optimization of the health insurance payment system.

## Methods

### Research design

This study employs the five-dimensional structure of the Consolidated Framework for Implementation Research (CFIR), which includes Innovation, Outer setting, Inner setting, Roles, Characteristics, and Process, to guide future implementation and evaluation. This comprehensive framework facilitates a structured and systematic approach to guiding and assessing policy execution, and it actively assists in identifying potential and actual factors that influence the progress and outcomes of implementation (23). By integrating a hierarchical exploration from the macro (senior hospital management) to meso (department and unit managers) to micro (medical, technical personnel, individual implementers) levels, we can gain a clearer understanding of how the DIP system disseminates information and develops signals to affect internal hospital decision-making and direction, thereby influencing operational models and reward and punishment mechanisms, ultimately manifesting in the policy cognition, strategic thinking, and medical behaviors of healthcare professionals.

Researchers collected perceptions and evaluations of implementing the Diagnosis-Intervention Packet (DIP) through semi-structured interviews with participants. By inductively coding the viewpoints of each interviewee across the five dimensions, we synthesized the interactions at different levels, elucidating how medical institutions implement policies and objectives through internal adjustments and coordination. We also explored individuals’ reactions and implementation status at the micro-level to management at the meso-level. From the acquisition and organization of this information, we identified the barriers and facilitators to the implementation of DIP.

### Study setting

In the study, we selected a large public hospital in Guangzhou, one of the first batch of tertiary-level hospitals in Guangdong Province, with a history of operation spanning over a century, making it representative. The research team conducted in-depth investigations within the medical institution, carrying out on-site field interviews with nine participants. Before the implementation of the field investigation, the team leader organized the division of labor among team members to collect and organize background information on the case hospital. The supervising instructor provided interview training for all personnel, standardizing the interview execution criteria and working group assignments.

The selection of interview subjects is based on the relevant departmental sections implementing DIP as a clue, combining the background of the hospital system, on-site tracking, and literature search. Full consideration was given to the work content of the personnel in different departments, the balance of their backgrounds, and the personal wishes of the respondents.

### Data collection

Between November 17 and November 30, 2023, 16 separate semi-structured qualitative interviews were conducted. To increase methodological rigor, this study used purposeful theoretical sampling to select interviewees from administrative and clinical departments directly related to DIP reform. Interviews were continued until theoretical saturation was reached, i.e., three consecutive interviews in which no new themes emerged. Two interviewers were assigned to one interview unit, and each interview lasted 45–60 min while being audio-recorded and transcribed offline. Electronic interview transcripts were backed up and transcribed in groups. A total of 16 valid interview texts were collected and organized, integrating one copy of each hospital background collection material and the DIP interview research guidebook.

### Data analysis

The 16 valid interview texts were cross-checked and cleaned by the four groups of interview unit staff and imported into NVivo.14.0 software by four data coders for manual organization and coding. In terms of coding consistency, two researchers with qualitative research backgrounds independently carried out the initial coding, and consensus was reached on the divergent parts through discussion and guided review by qualitative research experts to ensure the stability of the coding results. The coding process used CFIR as the main coding framework to summarize and arrange the qualitative texts by five dimensions and 36 domains. This enabled the researchers to take the qualitative, stand-alone texts and build them into a salient structure of positive and negative contributing factors to policy implementation. The distribution and frequency of the key domains were statistically analyzed as a reference point for comparison, and the factors were hierarchically categorized in conjunction with affective words and attitudes in the textual content. Triangulation was also used in this study to enhance reliability and validity. Interviews were cross-checked with relevant hospital documents and information (e.g., performance notices, implementation rules, etc.), and interviews with key informants were confirmed, thus strengthening the reliability and robustness of data interpretation.

The coding process identified 27 parent nodes and 83 child nodes across the five dimensions of CFIR 2.0. Parent nodes corresponded to core constructs (e.g., 5–6 nodes under Innovation, such as “Comparative Advantage” and “Complexity”), while child nodes were derived through granular thematic analysis (e.g., “Theoretical Complexity” and “Clinical Adaptability” under “Complexity”). Node density analysis revealed the highest concentration in *Inner Setting* (32%, primarily reflecting departmental collaboration [IC01] and resource allocation [H01]) and *Innovation* (28%, aligned with DIP policy design discussions [Ge01, CM01]). Inter-coder reliability was confirmed with Cohen’s *κ* = 0.78 (95%CI 0.72–0.84) based on dual independent coding of 30% transcripts.

### Ethical considerations

Ethical approval for this research was obtained from the First People’s Hospital of Guangzhou, with the approval number (K-2024-099-01). Before the interviews, the researchers assured the interviewees of the anonymization of their personal information.

## Results

### Summary of interviewees

We conducted semi-structured interviews with implementers of the in-hospital DIP medical payment reform in a locally representative public hospital, covering eight clinical medical departments (joint surgery, neurology, hematology, obstetrics, traditional Chinese medicine, pediatrics, geriatrics, and obstetrics, and gynecology) as well as six administrative functions (medical insurance office, medical record department, quality control department, economic management department, complaint management department, information center) covered a total of 16 implementers. Their basic information is described and organized in Table 1.

**Table 1:** Interviewed departments and interviewees included in November 2023.

Interview subject		Departments/Interviewees (%)
**Departments** (*n* = 14)	**Administrative departments** (Economic Management, Medical Records, Quality Control, Complaints Management, Information Center)	6(43)
	**Clinical departments** (Hematology, Geriatrics, Pediatrics, Obstetrics, Chinese Medicine, Neurology, Joint Surgery, Gynecology)	8 (57)
**Individuals** (*n* = 16)	**Responsibility**	
	Managerial staff	12 (75)
	Operational staff	4 (25)
	**Roles**	
	Administrative Staff	6 (38)
	Clinical staff	10 (62)

### Facilitators and barriers

The study was coded using the dimensions and corresponding domains of the Comprehensive Framework for Implementation Research (CFIR) as a reference, it analyzed the interview texts, distilling the five thematic results and disaggregating the facilitators and impediments to the implementation of DIP reform policies. The interview texts were analyzed, the five thematic results were distilled, and the facilitators and impediments to the implementation of DIP reform policies were disaggregated based on the qualitative textual results. The disaggregated results can be seen in Table 2.

**Table 2:** Factors that promote and hinder the implementation of DIP payment.

CFIR construct		CFIR domain	Determinants facilitator/Barrie/Mixed
Innovation		Effectiveness	Mixed
		Comparative advantage	Facilitator
		Trust	Facilitator
		Applicability	Mixed
		Complexity	Barrier
		Supporting policies	Facilitator
Outer setting		Important events (outer pressure)	Barrier
		Outer attitudes	Mixed
		Local policy conditions	Mixed
		Collaborative relationships	Facilitator
		Policy and Laws	Mixed
		Outer incentives (financial aid)	Facilitator
Inner setting		Team structure	Mixed
		Collaboration within the hospital	Facilitator
		Information sharing	Facilitator
		Culture	Mixed
		Urgency of reform	Facilitator
		Compatibility	Mixed
		Relative priority	Facilitator
		Incentive system	Barrier
		Goal alignment	Mixed
		Available Resources	Barrier
		Accessibility of knowledge and information	Facilitator
Individuals	Roles	Leaders, team members, supporters	Mixed
		Individual’s adaptability to reform	Barrier
		Individual abilities	Mixed
	Characteristics	Scope of individual power (available resources)	Barrier
		Individual motivation	Barrier
Implementation process		Teamwork	Facilitator
		Needs assessment	Barrier
		Environmental assessment	Mixed
		Planning	Facilitator
		Adjust strategy	Facilitator
		Mobilization	Barrier
		Execute	Mixed
		Reflection and evaluation	Facilitator

Facilitators to implementation were predominantly high (39%), with 22% barriers to implementation.

### Domain 1: Innovation

The dimension of innovation lies in exploring the differences between the Diagnosis-Intervention Packet (DIP) and previous medical insurance payment systems. Respondents assess the execution status of regarding effectiveness, comparative advantage, adaptability, applicability, and complexity. A positive factor is that respondents generally agree that the DIP reform policy is the most important policy at the current stage of the hospital in terms of medical insurance. Recognizing the policy’s importance leads to higher compliance and trust among implementers. The comparative advantage of DIP lies in controlling overall medical expenses to improve medical efficiency and resource utilization while also strengthening medical standards. Still, there are also concerns about its limitations on high-end medical care. The DIP reform still faces issues, such as the need for rational optimization of disease groupings.

The interviewees’ policy implementation assessments and opinions are coded, starting with the English abbreviation of the department or section, followed by 01 for department managers and 02 for medical staff.

- ***Ge01:** The impact of DIP payments on clinical care is currently evident and is arguably one of the most important factors. There is no doubt that DIP payments are dominant and that they play the role of the baton in clinical work.*
- ***CM01:** DIP is in line with clinical needs and use. It was originally designed to maximize savings at the national level and minimize unneeded tests and treatments.*
- ***EM01:** We are increasingly facing insurance reform, which is becoming increasingly significant. As a source of funding, this affects everything. Hospital management needs to be more sophisticated; managers face greater operational management costs and pressures.*
- ***MR01:** The DIP payment reform is a kind of innovation; the process of reform is more tortuous, but in the other, process of reform is more tortuous, but from another perspective, it is a forward and progressive.*
- ***COM01:** Medical insurance reform is reform and the most important policy at this stage. The most important aspect of medical insurance is ensuring basic coverage. Early preventive care and early intervention are also very important but rarely mentioned.*

### Domain 2: Outer setting

Outer setting factors pertain to the external impact events and societal collaborative relationships (policies, legal environment, public attitudes) that the DIP reform encounters. Regarding the facilitation of DIP reform implementation, there has been an intensified supervision and review of medical institutions by higher authorities following the policy’s enactment. For hospitals, this has led to heightened demands for refined management, necessitating adjustments in their management approaches to align with the standardized requirements of the DIP system regarding diagnosis, treatment, and cost control. Regarding collaborative facilitation, hospitals engage in feedback with higher medical insurance departments and share reform experiences with peer institutions. The resulting hindrances include increased operational management costs and pressures for hospitals, exacerbating the operational burden and leading to inefficiencies in policy execution at the departmental level.

- ***MI02:** The oversight and evaluation of DIP are primarily conducted through two methods: supervision by higher authorities and self-inspection; performance is assessed mainly by the hospital’s economic management department, with regulatory scrutiny becoming increasingly stringent.*
- ***IC01:** Our information center mainly provides active support for policy implementation, studying policy changes in conjunction with the construction and renovation of information systems, and engages in training and exchange activities with higher authorities.*
- ***H01:** Departments within the hospital report to the medical insurance department, which then feeds back to higher authorities; the timeliness of issue resolution depends on the specific circumstances. The degree of response to the reform and the pressure faced by different departments* var*ies, which is related to the characteristics of their business operations.*

### Domain 3: Inner setting

Inner setting factors emphasize the conduction and feedback mechanisms within hospital management, detailing how strategies are deployed, and policies are implemented at every level. Although the DIP payment reform has altered the payment method, it remains consistent with the hospital’s overall operational objectives. Respondents have indicated that the DIP reform acts as a “conductor’s baton” within the hospital, with internal adjustments made in response to policy learning, thereby promoting dynamic implementation. The hospital’s medical insurance department primarily handles policy training (combining online and offline approaches) and collecting feedback. Personnel performance and reward, and punishment systems are adjusted with DIP requirements and medical insurance funds. Implementation hindrances are reflected in the varying completion rates of DIP reform indicators across different departments and sections, due to real differences in disease categorization and diagnostic behaviors. This leads to significant differences in the adaptability of various departments to the reform.

Differences in the completion rates of the DIP indicators in different departments actually reflected the essential differences in their patient composition and treatment controllability. The interviews revealed that the hematology and geriatrics departments often faced greater challenges in implementing DIP indicators and insufficient DIP balances due to the complexity of patients’ conditions, the combination of multiple chronic diseases, and more uncontrollable treatment pathways, while joint surgery and Chinese medicine departments had better DIP matching and better implementation due to the relative stability of their disease types and the standardization of their treatment pathways. Furthermore, the DIP indicators are linked to personnel performance, departments with lower completion rates exhibit more pronounced emotional responses to the policy and, thus, are more prone to inefficiencies in execution.

- ***CM01:** Poor performance will lead to financial penalties. The prospect of deductions by medical insurance serves as the most primal motivation for us. Previously, the hospital primarily relied on a reward system, which led to a more cursory approach to work; now, there is increased attention to cost control and adherence to protocols.*
- ***MR01:** The hospital has appointed medical insurance specialists, also known as medical insurance administrators, in each department. These specialists form small teams with department heads to implement relevant policies. When conducting a detailed analysis of the operational status of departments, we employ a* var*iety of management tools. For instance, when analyzing disease types, we utilize the Pareto Principle, Alpha Analysis, and the Boston Matrix, along with various information systems.*
- ***EM01:** The performance assessment for our hospital staff has not changed significantly; it has been adjusted to accommodate and align with the existing performance and medical insurance cost systems.*
- ***Ge01:** The operational goals of the departments have not seen a drastic shift, but there is a greater emphasis on addressing the primary contradictions, moving away from the previous approach of conducting comprehensive examinations and treatments. This means that when facing patients with multiple coexisting conditions, the focus may be on resolving the most severe issue rather than managing all conditions simultaneously.*
- ***MR01:** The medical insurance office first understands the policies before arranging training for personnel in* var*ious departments. They regularly visit clinical departments to address queries. Our hospital conducts two to three internal audits per year, and they adopt multiple formats to train frontline staff.*

### Domain 4: Individuals

The individual dimension is bifurcated into the sub-dimensions of roles and characteristics. On the roles sub-dimension, emphasis is placed on the role of middle and senior managers within the organization. In the implementation of DIP, hospital administrators recognize its benefits as being closely intertwined with the hospital’s operational management, thereby leading and facilitating the execution of policies as opinion leaders. In the characteristics sub-dimension, the focus is on the adaptability and motivation of individual implementation units. The role of individual factors in promoting the implementation of DIP lies in enhancing the implementation personnel’s emphasis on disease diagnosis grouping and the completion of medical records. Interviewees involved in the DIP implementation widely accept that DIP reform is a process of gradual adaptation. Concurrently, regarding individual performance, different departments face varying pressures, with some interviewees indicating that the reform has increased the burden of clinical work, effectively hindering the implementation of individuals’ proactivity and the mobilization of individual available resources.

- ***EN01:** Our department staff always discuss whether DIP will restrict the development of new clinical technologies in clinical practice. Some departments have this concern because, in terms of business development, they employ new technologies, especially new materials, but are constrained by the impact of DIP payment.*
- ***Ge01:** The incentive direction of the reform may lead to polarization among physicians. When dealing with patients whose conditions are neither severe nor mild, medical staff may be reluctant to provide comprehensive medical services and consider the point value when choosing the primary diagnosis. This could lead to an inaccurate grasp of the patient’s actual condition, affecting treatment outcomes and patient satisfaction due to unmet medical needs. Medical staff can submit their opinions and suggestions through specific channels. However, the lack of a concrete mechanism to handle this feedback and advice on improvement measures is challenging.*
- ***MR01:** Our approach is primarily one of cooperation and support. But work pressure increases, and we can only go with the flow and enhance our capabilities.*
- ***H01:** The DIP reform has added a significant amount of extra workload to our department. Our department frequently encounters issues with cost settlements exceeding budgets, thus increasing the workload. Everyone can only do their best, but if the policies are too restrictive and cannot be met, doctors will have grievances. We believe that special cases should be resolved according to the actual situation. For example, it is not reasonable to deduct money from doctors for patients who can afford to pay for their medication out-of-pocket.*
- ***N01:** When clinical medical staff encounter unreasonable situations, such as uncontrollable, excessive-cost patients, apart from feeding back opinions to the department head and the medical insurance office, we can only choose to compromise and cooperate, adjusting our medical practices to follow the policy. The channels for feedback are few and singular.*

### Domain 5: Implementation process

Research on the implementation process serves to holistically delineate the execution and propagation pathways of the DIP policy (32). The implementation of the DIP reform is led primarily by the hospital’s medical insurance department, with administrative personnel responsible for oversight and self-auditing. Various departments within the hospital are tasked with policy implementation and upward feedback. The hospital integrates the DIP implementation framework into constructing its information systems to advance cost estimation, monitoring, and quality control. Adjustments to the reform’s implementation are made through the aggregation of departmental feedback and regular self-audits. This collaboration has effectively facilitated the control of medical insurance costs and the maintenance of financial balance. However, impeding factors are not to be overlooked, including inefficient execution due to unclear policies and undefined responsibilities within the hospital departments, and inaccurate needs assessment of implementing individuals, leading to some personnel failing to adjust promptly.

- ***MI02:** The medical insurance Bureau’s settlement is based on the whole hospital as a unit, the settlement of medical expenses, the balance of the hospital, and the amount of excess fees. The hospital’s management is subdivided into* var*ious departments.*
- ***EN01:** DIP reform has a neutral effect on hospital efficiency, depending on how the hospital is managed. Management in place will benefit the hospital, which means we must pay more attention to improving medical quality.*
- ***IC01:** Some of the medical insurance department’s requirements for the information system are not practical, such as the requirement that the system be improved in 5 days. In fact, the system cannot be changed in 20 days, and the change is more complicated. People who make policies do not understand information technology, often leading to unrealistic information requirements.*
- ***JS01:** In the early days, the hospital staff faced many problems and disagreements regarding DIP. Later, we gradually adapted and cooperated with the policy, improved the compliance degree of medical records, and progressively standardized them.*
- ***COM01:** The effect of policy implementation is also closely related to the understanding of patients and medical staff to the policy, and the actual change is not significant with the reform of medical insurance settlement.*
- ***QC01:** There are differences in implementation standards between departments. The medical insurance management department manages from the perspective of funds, and the medical management department considers the overall medical quality and medical process. There are some data differences in the process of implementing clinical pathways.*

## Discussion

Under the safeguarding impetus of national policy and platform construction, the reform of the DIP payment method is advancing steadily. Its leverage effect has effectively prompted medical institutions to gradually shift from extensive, expansionary operations to refined cost control and internal optimization (33). To date, the reform of medical payment methods has essentially achieved full coverage, and initial success is being seen in local practices. The next planning phase involves conducting practices with a more systematic and comprehensive depth and breadth (34).

Compared to most domestic and international scholars who focus on quantitative studies surrounding medical costs or hospital operational efficiency when researching the DIP payment reform, this study conducts a qualitative investigation into the internal response of a representative public tertiary hospital in the region to explore the status and challenges of the reform’s implementation in hospital operations. In contrast to previous studies, this research places greater emphasis on the responses of hospital staff to the DIP payment reform and its impact on policy implementation.

The synthesis of qualitative interview results indicates that, in the dimension of innovation, positive factors include the comparative advantages of the policy, its credibility, and supporting policies. In contrast, the complexity of the policy tends to hinder its implementation. Among outer setting factors, collaborative relationships and external incentives play a facilitating role, and significant events, as external pressure factors, impede policy implementation to some extent. From the inner setting dimension, active internal collaboration, information sharing, the urgency of reform, relative priority, and access to information and knowledge are conducive to implementing DIP innovation but also reveal a lack of internal incentive systems and available resources within the hospital. From an individual perspective, the adaptability of the implementing units to reform and their access to resources and scope of authority are somewhat limiting in policy implementation. In terms of the overall dimension of the implementation process, the effective execution of teamwork, planning, strategy adjustment, feedback, and evaluation is beneficial in demonstrating positive policy outcomes. However, there is still a need to pay attention to assessing individual needs and mobilization.

The DIP payment is an innovative measure in payment reform; its innovative design and empirical basis are crucial to the success of its implementation (35). Observations from field investigations indicate that external environmental factors such as policy, economy, and patient demand influence the implementation of the DIP payment method. Internal factors within the hospital, including organizational culture, resources, and system management, significantly impact the acceptance and enforcement of the DIP payment method (36). At the level of individual roles and characteristics, the cognition, capabilities, and attitudes of medical staff toward the reform affect the actual effectiveness of policy advancement. The overall implementation process, encompassing planning, strategy development, execution, and confirmation, requires the active participation of hospital management and medical personnel.

Overall, implementing the DIP reform has achieved positive effects in reducing the medical burden on the population, guiding the allocation of medical resources, and standardizing diagnostic and treatment practices (37). The reform of the DIP payment method in public hospitals, supported and supervised by national policies, is progressing gradually but still faces multifaceted challenges. Through analysis using the CFIR framework, hospital administrators and policy researchers can more systematically identify and address these challenges, facilitating the smooth implementation of the DIP payment reform.

The findings of this study echo the international experience with the implementation of value-oriented payment models such as disease-based payment (DRG) or packaged payment. Since the implementation of the G-DRG system in Germany in 2004, it has been noted that while controlling healthcare costs, the system has triggered controversies about limited clinical autonomy, especially in departments with diverse treatment modalities and complex patient conditions (38). France’s performance-oriented inpatient payment reform, while improving efficiency, also faced problems with “diagnostic upcoding” behavior and insufficient attention to patients with slow and complex diseases (39). Similarly, in the United States, the BPCI-A (Bundled Payments for Care Improvement-Advanced) program, while effective in reducing healthcare expenditures in some departments (orthopedics, cardiology, etc.), has also reduced the focus on access to care for high-risk patients (40).

Although China’s DIP reform is a homegrown original payment model, its core challenges are highly common to the countries mentioned above. DIP’s classification of disease groups based on cost homogeneity may be insufficient to adequately reflect clinical complexity, which, in turn, may lead to physicians’ tendency to engage in harm avoidance behaviors. These responses may affect the quality of patient-centered care, especially for the older adults, multimorbid coexisting individuals, and lower socioeconomic groups (41).

From an ethical perspective, payment reforms such as DIP need to strike a balance between cost efficiency and equity of access. International experience suggests that one-size-fits-all cost containment may exacerbate inequalities in access to healthcare if there is a lack of contextual flexibility. Therefore, policymakers should consider introducing approaches such as “risk adjustment mechanisms,” “high-complexity elasticity bands,” and patient-reported outcomes (PROs) to improve the system’s responsiveness to patients’ actual needs (42). Some regions in China have already begun to pilot reforms - for example, Zhejiang Province’s 2024 DIP reform program has included PRO data in 10% of the payment score, reflecting an initial exploration of DIP fairness calibration (43).

Faced with the tension between cost containment and clinical autonomy, DIP should increase its flexibility. The French T2A system can be modeled on the “clinical exception clause” that protects physicians’ professional judgment, while Germany requires the recording of patient-physician shared decision-making in the G-DRG system (44).

To address the identified challenges and support the long-term implementation of DIP payment reform, the following multi-tiered strategies are proposed, structured around three levels: hospital-based adaptation, payment system refinement, and national strategy integration.

### Hospital management optimization recommendations (short term, 1–3 years)

Optimize performance incentives:

Interviews showed that the implementation of the DIP payment reform may lead to behavioral adjustments when doctors face complex cases (Domain 3 CM01), affecting the quality of treatment. It is recommended that a “fault tolerance zone” be introduced into the performance appraisal to alleviate the subjective pressure on doctors and improve the acceptability and efficiency of the policy.

Implementing differentiated assessment weights:

There are differences in the adaptability of different departments in the DIP payment reform, and uniform assessment standards may lead to greater pressure on certain departments. Thus, Differentiated assessment weights should be implemented according to the actual situation, and flexible thresholds (e.g., ±15%) should be adopted for special departments such as geriatrics and oncology to avoid “one size fits all.

### Suggestions for improving the medical reform system (medium-term 3–5 years)

Dynamic adjustment mechanism for disease grouping:

Jointly establish expert committees on DIP grouping at the level of diagnostic and treatment institutions at all levels with clinical, medical insurance, and case departments to review and adjust disease groupings on a regular basis in order to gradually optimize the rationality of the groupings.

Strengthening financial support for information technology construction:

The effective operation of the DIP payment system relies on high-quality medical data and information systems. However, medical institutions are under both financial and technological pressure to build informatization (Domain 5IC01 Policymakers do not understand informatization needs) It is recommended that the government increase financial investment in hospital informatization construction to improve data collection and disease identification capabilities.

### National strategy-oriented recommendations (long-term more than 5 years)

DRG/DIP synergistic development:

Given that DRG is applicable to acute diseases and short-term hospitalization, and DIP is applicable to chronic diseases and multiple treatments, it is recommended to establish a “categorized and segmented payment mechanism,” adopting DRG in the acute stage and DIP for chronic disease management, so as to adapt to the needs of different service pathways and payment efficiencies.

Enhancing patient participation:

The current DIP assessment mechanism is dominated by the medical service provider, and it is recommended to introduce patient cost satisfaction survey data as a payment correction factor, and to explore the doctor-patient balance sharing mechanism to enhance the public acceptance of the policy and the sense of fairness.

## Limitations

This study also has its limitations. Firstly, the research was conducted solely within one medical institution without the introduction of a control group. Thus, it is not possible to rule out the possibility that certain specific factors may alter the behavior of patients and the hospital. Secondly, due to the inherent limitations of qualitative research, the number of subjects is small, which includes the potential risk of selection bias. The subjective responses of the subjects may not accurately reflect the true situation. Thirdly, this study did not involve patient visits, and patient feedback on the reform, which is the ultimate indicator of implementation effectiveness, was not included. Additionally, there may be a degree of social desirability bias among the administrative and clinical staff interviewed when talking about sensitive or performance-related issues such as health care reform, and some of the interviewees may be inclined to express “positive attitudes” consistent with the policy rather than fully revealing the reality of the difficulties or their realities. To a certain extent, this limits the insights of this study on the individual dimension. Future research may consider combining anonymous questionnaires and third-party observation to reduce such bias. Meanwhile, the study population on which the conclusions of this paper are based is a tertiary public hospital in Guangzhou City, and its results still need to be treated with caution when generalized to other regions or different levels of healthcare institutions.

However, the results of this study are still representative and reliable; the management case of DIP in public hospitals and the interview data formed can reflect the internal response strategies and transmission pathways within the hospital, still providing a multi-dimensional perspective for exploring subsequent optimization of the reform. As the reform process deepens and policies are updated, further assessment of the long-term impact and overall effectiveness of DIP will be needed in the future.

## Conclusion

Our results highlight the key facilitators of the implementation of DIP payment in hospitals: the emphasis placed on DIP reform by hospital leadership, high compliance among implementing personnel, smooth communication with higher-level medical insurance departments and other hospitals, and the establishment of an information-based measurement and quality control system adapted to the DIP framework. The main obstacles to implementation include restrictions on high-end medical care, the need to optimize disease groupings, high demands for refined hospital management, and increased burden on clinical work. Additionally, due to the linkage of physician performance incentives to the surplus medical insurance funds, there is a significant variation in the adaptability of different departments within the hospital to the DIP reform. Currently, under the DIP payment reform, the hospital has been effective in controlling overall medical insurance costs, achieving a balance between income and expenditure.

## Data Availability

The raw data supporting the conclusions of this article will be made available by the authors, without undue reservation.
